# Linac-based stereotactic radiotherapy and radiosurgery in patients with meningioma

**DOI:** 10.1186/1748-717X-9-78

**Published:** 2014-03-20

**Authors:** David Kaul, Volker Budach, Reinhard Wurm, Arne Gruen, Lukas Graaf, Piet Habbel, Harun Badakhshi

**Affiliations:** 1 Department of Radiation Oncology, Charité School of Medicine and University Hospital, Campus Virchow-Klinikum, Augustenburger Platz 1, 13353 Berlin, Germany; 2 Department of Oncology and Hematology, Charité School of Medicine and University Hospital, Campus Mitte, Charitéplatz 1, 10117 Berlin, Germany; 3 Department of Radiation Oncology, Hospital Frankfurt/Oder, Müllroser Chaussee 7, 15236 Frankfurt, Germany

**Keywords:** Meningioma, Local control, Stereotactic fractionated radiotherapy, Image-guided radiotherapy

## Abstract

**Background:**

It was our purpose to analyze long-term clinical outcome and to identify prognostic factors after Linac-based fractionated stereotactic radiotherapy (Linac-based FSRT) and stereotactic radiosurgery (SRS) in patients with intracranial meningiomas.

**Materials and methods:**

Between 10/1995 and 03/2009, 297 patients with a median age of 59 years were treated with FSRT for intracranial meningioma. 50 patients had a Grade I meningioma, 20 patients had a Grade II meningioma, 12 patients suffered from a Grade III tumor, and in 215 cases no histology was obtained (Grade 0). Of the 297 patients, 144 underwent FSRT as their primary treatment and 158 underwent postoperative FSRT. 179 patients received normofractionated radiotherapy (nFSRT), 92 patients received hypofractionated FSRT (hFSRT) and 26 patients underwent SRS. Patients with nFSRT received a mean total dose of 57.31 ± 5.82 Gy, patients with hFSRT received a mean total dose of 37.6 ± 4.4 Gy and patients who underwent SRS received a mean total dose of 17.31 ± 2.58 Gy.

**Results:**

Median follow-up was 35 months. Overall progression free survival (PFS) was 92.3% at 3 years, 87% at 5 years and 84.1% at 10 years. Patients with adjuvant radiotherapy showed significantly better PFS-rates than patients who had been treated with primary radiotherapy. There was no significant difference between PFS-rates of nFSRT, hFSRT and SRS patients. PFS-rates were independent of tumor size. Patients who had received nFSRT showed less acute toxicity than those who had received hFSRT. In the Grade 0/I group the rate of radiologic focal reactions was significantly lower than in the atypical/malignant histology group.

**Conclusion:**

This large study showed that FSRT is an effective and safe treatment modality with high PFS-rates for intracranial meningioma. We identified “pathological grading” and and “prior surgery” as significant prognostic factors.

## Background

Meningioma is the second most common primary intracranial tumor that arises from the cap cells of the arachnoid membrane and is more common in women than in men, incidence peaks in the fifth, sixth, and seventh decade [1]. About 85% of meningiomas are slow growing Grade I tumors, approximately 10% are Grade II atypical lesions, and 3–5% are anaplastic Grade III lesions.

Microsurgical resection is usually the treatment of choice for symptomatic tumors but may not always be possible, due to proximity to critical structures. Higher Grade tumors have higher risks of recurrence after surgical resection [2,3]. Postoperative FSRT may prolong time to recurrence and is usually recommended after incomplete resection. Inoperable symptomatic Grade I meningiomas can be treated using FSRT with results comparable to those of complete resection [4,5].

Grade II meningiomas are found in 5-20% of the patients; 40-60% of these patients remain disease-free at 10 years after definitive treatment [6,7]. Grade III tumors account for 1-5% and recurrence-free survival is usually less than 2 years [7]. Grade II/III meningiomas are commonly treated with postoperative FSRT after incomplete or complete resection [8,9]. In the present study, we analyzed the results in one of the largest populations of meningioma-patients treated with Linac-based image-guided FSRT in a single institution. The purpose was to gain information on long-term clinical outcome, relevant prognostic factors and contribute to the ongoing multidisciplinary discussions.

## Methods

### Treatment decisions, patient selection and dose regimens

We performed a retrospective analysis of 372 patients who underwent FSRT of an intracranial meningioma between 10/1995 and 03/2009. 5 patients receiving re-irradiation due to a secondary meningioma were excluded. In 3 patients the diagnosis was questionable, 62 patients were excluded because of incomplete follow-up. In 5 patients the fractionation scheme was not determinable. Follow-up data were analyzed until March 2010. The study was approved by the local Ethics Committée of Charité University Medicine, Berlin. The research was in compliance with the Helsinki Declaration.

In our institution treatment decisions are based on an interdisciplinary vote. Adjuvant FSRT is offered to all resected Grade II and III meningioma-patients; Symptomatic Grade I meningiomas are treated with adjuvant RT only after incomplete resection or when recurrence occurs after total resection. Inoperable Grade II or III tumors as well as inoperable symptomatic Grade I tumors are treated using primary FSRT.

1.6-2.2 Gy were considered normofractionated (nFSRT), 2.2-5 Gy were considered hypofractionated (hFSRT) and high single doses delivered in less than 5 sessions were considered stereotactic radiosurgery (SRS).

Tumors in close proximity to critical structures were assigned to nFSRT, while large tumors (> 2 cm) distant to critical structures underwent hFSRT and small tumors (< 2 cm) were treated by SRS.

### Stratification and variables

Patient data were analyzed according to grading, location, predicted peri-operative risk/operability, tumor size and sequence of therapy. Two groups were defined: group 1 encompasses all Grade I meningiomas, as well as all meningiomas with no histology available (Grade 0). Group 2 encompasses all Grade II and III meningiomas.

The tumor location was divided into 3 groups according to the Novel “CLASS” Algorithmic Scale for Patient Selection in Meningioma Surgery: low risk, medium risk, high risk and optical nerve sheath (ONSM) [10].

Follow-up examinations, including MRI as well as clinical, and neurologic examinations were performed at 6 weeks, 3 months, 9 months, 15 months after treatment and then annually.

We distinguished between primary radiation treatment and postoperative radiotherapy. Acute toxicity in the first 90 days after FSRT was graded using a modified version of the Common Terminology Criteria of Adverse Events (CTCAE v4.0).

### Technical set-up

From 1995–2003 meningioma patients underwent “sharp” fixation using a stereotactic head ring and an oral bite Plate. A 6 MV Linac (Varian® USA) with an add-on micro-multileaf collimator (mMLC) (BrainLab® Co, Germany) was used. Coordinats for SRS were set by a laser-based stereotactic localizer. This set up allowed delivering shaped beams. In 2004 we started using Novalis® (BrainLab®) with beam shaping capability using build-in MLC and image guidance. Novalis ExacTrac® image guided frameless system enabled us to image the patient at any couch position using a frameless positioning array. MRI/CT-fusion planning was performed. The three-dimensional treatment planning system Brainscan® (Brain Lab AG, Germany) was used, which was later replaced by iplanRT®. The gross tumor volume (GTV) was defined as the area of contrast enhancement on T1-weighted MRI images, the planning target volume (PTV) included a 2 mm isotropic safety margin. The dose was prescribed to a reference point, which was the isocenter (or the center of GTV), though 100% was not the maximum dose but the dose at the aforementioned reference point. Patients received the prescribed dose to the 80th isodose at the tumor margin plus a safety margin of 5 mm in Grade II and III meningiomas. Organs at risk (OAR) such as optic nerves, the chiasm, lenses and the brainstem, were delineated. Dose constraints were according to the data published by Emami et al. 1991 [11]. The TD 5/5 to be respected was as follows: for optic nerves 50 Gy, for chiasm 50 Gy, for lenses 10 Gy and for the brainstem 50 Gy, respectively.

### Formulas and statistics

The equivalent 2 Gy dose (EQD2) was calculated according to the formula,EQD2 = n × d × (a/b + d)/(a/b + 2).

All statistical analyses were performed using IBM SPSS Statistics 19 (New York, USA).

## Results

### Patients

297 meningioma patients treated in our department between October 1995 and March 2009 were included in the analysis. Patient characteristics are summarized in Table 1.

**Table 1:** Characteristics of the 297 meningioma patients analyzed

		**Overall collective**		**Group 1**		**Group 2**	
		**(n = 297)**		**U/WHO1**		**WHO2/3**	
				**(n = 265)**		**(n = 32)**	
		**Mean**	**Min/Max**	**Mean**	**Min/Max**	**Mean**	**Min/Max**
Age with beginn of RT		59	20/87	59	20/87	60.13	38/76
Mean volume		15.01	0.26/190.85	15.77	0.26/190.85	6.62	0.84/27.42
		**n**	**%**	**n**	**%**	**n**	**%**
Gender	m	95	32	75	28.3	20	62.5
	f	202	68	190	71.7	12	37.5
Location	Skull Base	254	85.5	246	92.8	8	25
	Falx/Parasagittal	20	6.7	10	3.8	10	31.3
	Convexity	23	7.7	9	3.4	14	43.8
WHO grading	n/a	215	72.4	215	81.2	-	-
	WHO 1	50	16.8	50	18.9	-	-
	WHO 2	20	6.7	-	-	20	62.5
	WHO 3	12	4.0	-	-	12	37.5
Prior surgery	Primary RT	144	48.5	143	54	1	3.1
	Adjuvant RT	153	51.5	122	46	31	96.9
Peritumoral edema	yes	13^§^	6.6	12^Ÿ^	6.7	1	5.56^$^
Multiple meningiomas	yes	58	19.5	52	19.6	6	18.8
Fractionation Scheme	FSRT	179	60.3	158	59.6	21	65.6
	hFSRT	92	31.0	82	30.9	10	31.3
	SRS	26	8.8	25	9.4	1	3.1
Mean total dose		47.7	13.5/95.4	47.1	13.5/63	52.4	15/95.4
Follow up-time in months		35	1/132	38	1/132	12.5	2/80

^§^n = 197.

^Ÿ^n = 197.

^$^n = 18.

#### Localization

Tumors were grouped according to the “CLASS” Algorithmic Scale for Patient Selection in Meningioma Surgery: 9.8% had a low risk localization, 13.5% showed a moderate risk localization, 71.7 showed s high risk localization and 5.1 percent were ONSM.

### Progression free survival

**Figure 1:**
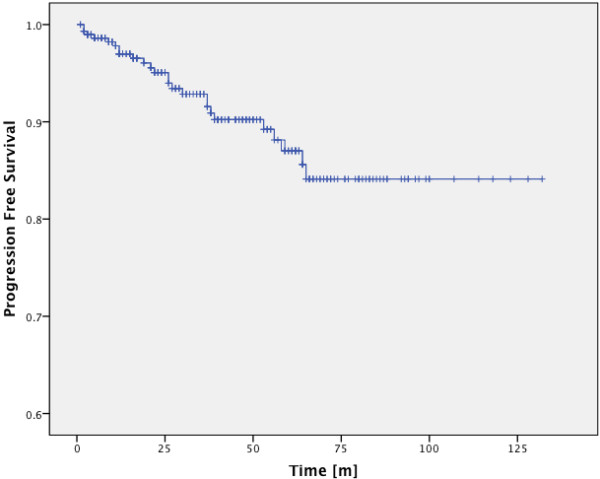
PFS-rates of the entire cohort.

PFS-rates are summarized in Table 2. In the entire cohort progression free survival was 92.3% after 3 years, 87% after 5 years and 84.1% after 10 years (Figure 1).

**Table 2:** PFS-rates

	**PFS-rates**		
	**3 years**	**5 years**	**10 years**
Entire cohort	92.30%	87.00%	84.10%
Group 1 (unknown/Grade I)	96.80%	92.70%	89.60%
Group 2 (Grade II/III)	52.60%	19.70%	n/a
Unknown histology	97.40%	94.20%	90.40%
Grade I	93.90%	85.80%	85.80%
Grade II	62.70%	41.80%	n/a
Grade II	29.30%	n/a	n/a
Volume < 60 ccm	95.80%	91.20%	89.30%
Volume > 60 ccm	100%	85.70%	n/a
Primary RT	98.10%	98.10%	96.80%
Postoperative RT	87.60%	78.10%	73.40%
nFSRT	92.70%	88.90%	86.90%
hFSRT	92.40%	80.90%	n/a
SRS	95.80%	87.80%	n/a

**Figure 2:**
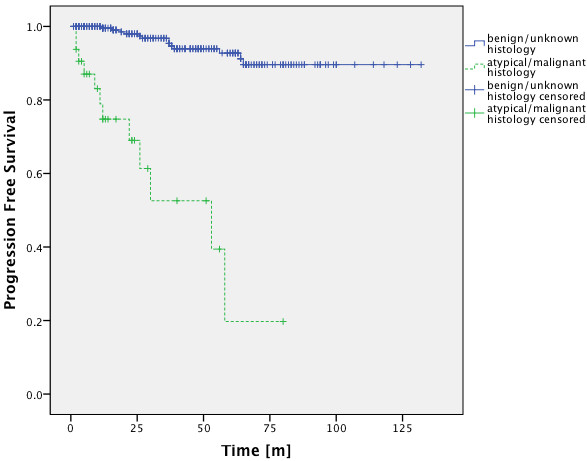
**PFS-rates of group 1 and group 2.** There is a significant difference between the PFS-rates of both groups (p < 0.001; Log-Rank Test).

**Figure 3:**
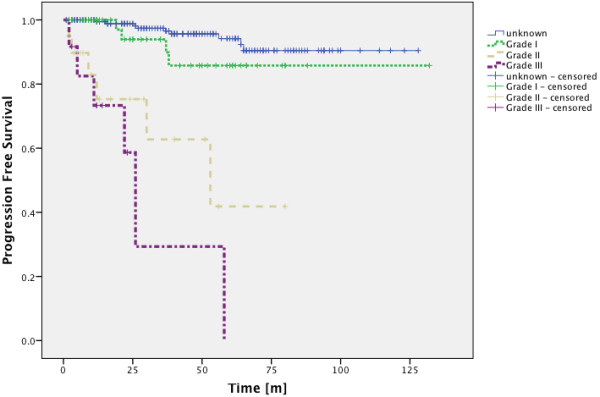
**PFS-rates for meningiomas of unknown histology as well as GradeI I, II and III meningiomas.** There is no significant difference between the PFS-rates of meningiomas of unknown histology and Grade I tumors (p = 0.172; Log-Rank Test). Unknown histology vs. Grade II (p < 0.0001; Log-Rank Test), unknown histology vs. Grade III (p < 0.0001; Log-Rank Test), Grade I vs. Grade II (p < 0.002; Log-Rank Test), Grade I vs. Grade III (p < 0.0001; Log-Rank Test).

In the benign/unknown histology group PFS-rates were 96.8%, 92.7% and 89.6% at 3, 5 and 10 years (Figure 2). Patients with histologically proven Grade I meningioma showed PFS-rates of 93.9% after 3 years and 85.8% after 5 years and 10 years and patients with unknown histology showed PFS-rates of 97.4%, 94.2% and 90.4% for 3, 5 and 10 years (Figure 3), there was no significant difference between PFS-rates of proven Grade I tumors and tumors of unknown histology (p = 0.172, Log-Rank Test). In the Grade II/III group PFS-rates were 52.6% after 3 years and 19.7% after 5 years, 10 year rates could not be calculated since all cases had been censored (Figure 2). The difference in PFS-rates between group 1 and group 2 was highly significant in the Log-Rank Test (p < 0.0001). Patients with Grade II meningioma had PFS-rates of 62.7% after 3 years and 41.8% after 5 years, 10 year rates could not be calculated since all cases had been censored (Figure 3).

**Figure 4:**
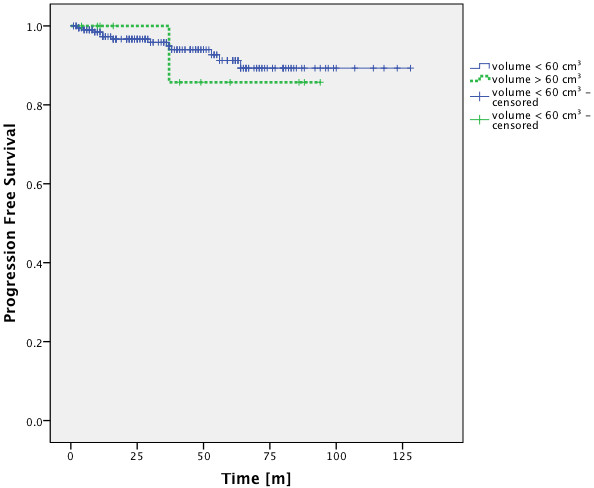
**PFS-rates of patients with tumors above and below 60 cm**^**3**^**.** There is no significant difference between the PFS-rates of patients with tumors above and patients with tumors below 60 cm^3^ (p = 0.768; Log-Rank Test).

Patients with a target volume of more than 60 cm^3^ have been described to have significantly worse local control rates [12]. In this study tumors with a volume below 60 cm^3^ had no significantly better PFS-rate than those over 60 cm^3^ (Figure 4). It has to be mentioned though, that there is a bias in treatment selection, since tumors with a diameter < 2 cm usually received SRS.

**Figure 5:**
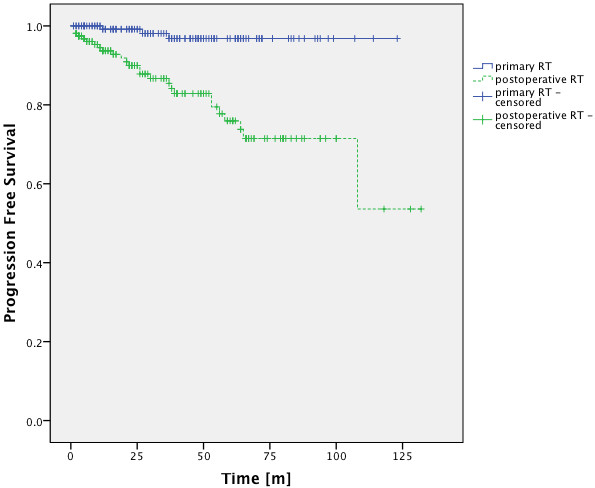
**PFS-rates for patients with primary and postoperative FSRT.** There was a significant difference in PFS-rates for patients treated with primary and adjuvant FSRT (p < 0.001 Log-Rank Test).

An analysis of the factor “prior surgery” showed significant differences in PFS-rates. Patients who had undergone surgical resection showed worse PFS-rates (Figure 5).

**Figure 6:**
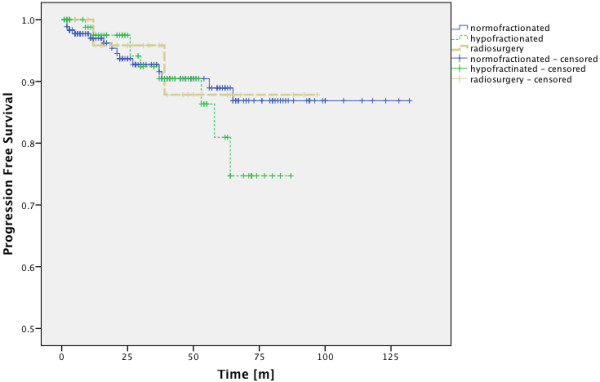
**PFS-rates for patients with normofractionated, hypofractionated and radiosurgical treatment.** There was no significant difference between the 3 groups (p = 0.811; Log-Rank Test).

Patients with nFSRT received a mean total dose of 57.31 ± 5.82 Gy, patients with hFSRT received a mean total dose of 37.6 ± 4.4 Gy and patients who underwent SRS received a mean total dose of 17.31 ± 2.58 Gy. These values translate into EQD2_10_-values of 56.37 ± 5.72 Gy for nFSRT, 45.17 ± 3.26 Gy for hFSRT and 38.93 ± 9.66 Gy for SRS. There was no significant difference in terms of PFS-rates between nFSRT, hFSRT and SRS treatment. the 3 groups (p = 0,811; Log-Rank Test) (Figure 6).

### Radiologic response

Radiologic response was evaluated using MRI (T1, T2 and FLAIR sequence). Response rates are shown in Table 3. In group 1 252 patients (95.1%) were locally controlled, 18.1% tumor regression. Only 4.9% of patients showed local progression. In the atypical/malignant histology group worse local control was seen (62.5%, p < 0.001), but interestingly there was no significant difference between group1 and group 2 when looking at tumor regression (18.1% in group 1 vs. 15.6% in group 2). In our analysis of subgroups none of the following variables showed significant influence on tumor regression, neither “tumor volume” nor “prior surgery” nor the fractionation scheme.

**Table 3:** Tumor response rates

		**Tumor response**			**Total**
		**Progress**	**Stable**	**Regression**	
Group 1	n	13	204	48	265
	%	4.9%	77%	18.1%	100.0%
Group 2	n	12	15	5	32
	%	37.5%	46.9%	15.6%	100.0%
Overall collective	n	25	219	53	297
	%	8.4%	73.7%	17.8%	100.0%

### Acute toxicity

The most common acute Grade I symptoms for the entire cohort were headache, fatigue, local alopecia and local skin reaction. The most common acute Grade II symptoms were headache, vertigo and nausea.

The difference in acute toxicity between group 1 and group 2 was not significant.

Patients with tumors smaller than 60 cm^3^ did not show a significantly different rate of acute toxicity than patients with a tumor diameter above 60 cm^3^ (n = 192, p = 0.28). As mentioned above though there is a bias in treatment , since smaller tumor usually received SRS.

There was a significant difference in acute toxicity for different fraction schemes: Patients receiving nFSRT showed more acute toxicity compared to hFSRT or SRS (67.1% vs. 47.9% and 35%). This difference was mainly due to Grade I reactions (FSRT: 50.3%, hFSRT: 31% SRS: 10%; p < 0.001), Grade II and III reactions were even a bit higher in Patients receiving hFSRT or SRS, although this effect was not significant.

### Late toxicity

For n = 276 patients (92.9%) of the entire cohort evaluation of late toxicity was available, 98 (35.5%) showed late toxicity. The most common Grade I symptoms were headache and fatigue. The most common Grade II symptoms were headache, dizziness and seizures. Grade III late toxicity was seen 0.7% of the patients.

The difference in late toxicity between group 1 and group 2 was not significant.

### Focal reactions and corticosteroid treatment

New peritumoral edema during follow-up was described in 14.8% of all patients in the entire cohort. In group 1 the rate of focal reaction was significantly lower than in group 2; 12.8% compared to 31.2% (p = 0.014).

Therapy with corticosteroids was necessary in 12.8% of the patients. There was no significant difference between group 1 and group 2 concerning treatment with steroids (16.2% vs. 13.3%).

## Discussion

This study is the second largest analysis of Linac-based FSRT for intracranial Grade I-III meningiomas after the study by Milker-Zabel et al. [13]. We show here that Linac-based FSRT is a safe option in the treatment of intracranial meningiomas with a low risk of acute or late toxicity and can thus corroborate the findings by Milker-Zabel et al. Patients with meningiomas that score high on the “CLASS” -scale are more likely to receive primary radiotherapy meaning that the patients have undergone negative preselection. Considering this fact the good PFS rates seen after radiotherapy are even more encouraging.

In the entire cohort (Grade 0-III) progression free survival was 92.3% at 3 years, 87% at 5 years and 84.1% at 10 years. Similar results have been shown for gamma knife radiosurgery of Grade I-III meningiomas by Nakaya et al. who found PFS-rates of 94%, 83%, and 58% at 3, 5 and 10 years respectively [14].

In the benign/unknown histology group PFS-rates were 96.8%, 92.7% and 89.6% at 3, 5 and 10 years. In a study by Compten et al. similar PFS-rates for FSRT-treated benign and unknown histology meningiomas are shown: 96.3% at 3 years and 93% at 5 years [15].

Only looking at patients with histologically proven Grade I meningioma PFS-rates were 93.9% at 3 years and 85.8% at 5 years and at 10 years. Milker-Zabel et al. found similar PFS-rates of 98.5% at 3 years, 90.5% at 5 years, and 89% at 10 years for FSRT-treated Grade I meningiomas. In a study by Debus et al. the overall actuarial survival rate of patients with WHO Grade I meningioma was 97% at 5 years and 96% at 10 years [16]. Kreil et al. showed similar results for benign histology meningiomas treated with gamma knife radiosurgery: 98.5% at 5 years and 97.2% at 10 years [17].

We found that group 2 had PFS-rates 52.6% at 3 years and 19.7% at 5 years, 10 year rates could not be calculated since all cases had been censored. Compten et al. showed a slightly lower PFS-rate of 40% for GradeII/III meningiomas at 3 years [15].

Patients with Grade II meningioma had PFS-rates of 62.7% at 3 years and 41.8% at 5 years, 10 year rates could not be calculated since all cases had been censored. Milker-Zabel et al. showed better recurrence-free survival rates of 96% at 3 years, 89% at 5 years, and 67% at 10 years for Grade II meningiomas [12]. However these data might might be a statistically biased due to the small number of patients with proven Grade II meningioma.

In general it must be mentioned that the role of adjuvant radiotherapy of Grade II/III meningiomas remains unclear due to inconsistent evidence from retrospective studies [18].

Independent of histology we showed 5 year PFS-rates of 96.8% for patients who had undergone primary radiation and 78.1% for patients who had received surgical resection. Nutting et al. found a 5-year PFS-rate of 92% in patients with benign meningiomas which had been treated with adjuvant conventional FSRT after surgery [19]. In a study by Goldsmith et al. a 5-year progression-free survival rate of 89% was reported for patients with subtotally resected Grade I meningioma after postoperative FSRT [20].

We found a significant difference in PFS-rates for patients who had undergone surgical resection and those who had received primary radiation (p < 0.001, log-rank test). This result was to be expected since Grade I meningiomas are usually not treated with adjuvant FSRT after complete resection.

Milker-Zabel et al. found that patients with a tumor volume >60 cm^3^ had a higher recurrence rate than patients with a lower tumor volume. We did not find significant differences in PFS-rates between these two groups. However, considering that only 11 of the entire cohort showed a tumor volume above 60 cm^3^, the number of cases analyzed here might just be to low to be significant.

We found that patients with nFSRT showed more acute toxicity than those with hFSRT or SRS. This difference was mainly due to Grade I reactions. The rate of Grade II and III reactions was higher in patients who received hFSRT or SRS (however this effect was not significant). This might be due to the prolonged treatment in nFSRT-patients. These patients had the time to develop Grade I reactions whereas hypofractionated patients might have skipped Grade I reactions and developed Grade II/III reactions faster.

In the entire cohort 35.5% of the patients showed late toxicity. Compten et al. found late toxicity in 20.8% of meningioma patients after FSRT. Hamm et al. found late toxicity of only 14.3% in a cohort of 224 patients (183 nFSRT, 30 hFSRT and 11 SRS). However it has to be noticed that retrospective analyses of toxicity are bias-prone due to interobserver variabilities. Also the relatively high total dose delivered may play a role here.

Engenhart et al. who found 30% of patients to show chronic late toxicity after a mean single fraction dose of 29 Gy which is similar to 36% late toxicity found in SRS patients in this study [21].

We found Grade III late toxicity in 0.7% of the patients, which is similar to the results shown in a study by Goldsmith et al. where 3.6% of 140 patients treated with a radiation dose of 57.6 Gy showed Grade III late toxicity [20]. Jalali et al. found chronic late toxicity in 6% of 67 patients [22].

In our study new peritumoral edema was described in 14.8% of all patients in the entire cohort, slightly worse results were found in a study by Chang et al., where radiologic complications were seen in 23.6% of 179 patients treated with gamma knife radiosurgery after a mean follow-up of 37.3 months [23].

## Conclusion

The good long-term clinical outcome of FSRT in regard to its high effectiveness and low morbidity is indicating that it should be considered as an equivalent treatment option complementary to microsurgery and gamma knife radiosurgery.

## Competing interests

All authors confirm that there is no conflict of interests.

## Authors’ contributions

DK performed statistical analysis, wrote the manuscript, and supervised the discussion of the manuscript, HB planned experiments and took part in the discussion of the manuscript, LG collected data and performed statistical analysis, PH, AG and VB took part in the discussion of the manuscript, RW planned experiments. All authors read and approved the final manuscript.
